# Development of Digital Biomarkers of Mental Illness via Mobile Apps for Personalized Treatment and Diagnosis

**DOI:** 10.3390/jpm12060936

**Published:** 2022-06-06

**Authors:** I-Ming Chen, Yi-Ying Chen, Shih-Cheng Liao, Yu-Hsuan Lin

**Affiliations:** 1 Department of Psychiatry, National Taiwan University Hospital, Taipei 100, Taiwan; b90401022@gmail.com (I.-M.C.); jenniferchen98@gmail.com (Y.-Y.C.); scliao@ntu.edu.tw (S.-C.L.); 2 Department of Psychiatry, College of Medicine, National Taiwan University, Taipei 100, Taiwan; 3 Institute of Population Health Sciences, National Health Research Institutes, 35 Keyan Road, Zhunan, Miaoli County 350, Taiwan; 4 Institute of Health Behaviors and Community Sciences, College of Public Health, National Taiwan University, Taipei 100, Taiwan

**Keywords:** digital biomarkers, digital phenotyping, mobile applications, digital footprint, e-mental health

## Abstract

The development of precision psychiatry is largely based on multi-module measurements from the molecular, cellular, and behavioral levels, which are integrated to assess neurocognitive performances and clinically observed psychopathology. Nevertheless, quantifying mental activities and functions accurately and continuously has been a major difficulty within this field. This article reviews the latest efforts that utilize mobile apps to collect human–smartphone interaction data and contribute towards digital biomarkers of mental illnesses. The fundamental principles underlying a behavioral analysis with mobile apps were introduced, such as ways to monitor smartphone use under different circumstances and construct long-term patterns and trend changes. Examples were also provided to illustrate the potential applications of mobile apps that gain further insights into traditional research topics in occupational health and sleep medicine. We suggest that, with an optimized study design and analytical approach that accounts for technical challenges and ethical considerations, mobile apps will enhance the systemic understanding of mental illnesses.

## 1. Introduction

Diseases attributed to unhealthy behaviors and psychiatric disorders have been steadily on the rise in the past few decades. Yet, the surveillance for these health outcomes has remained largely unchanged. Surveys are still commonly used to assess people’s health, despite being inherently restrictive due to respondents’ reluctance to participate, social desirability biases, and recall biases. Furthermore, the substantial cost required to conduct surveys means that many topics cannot be adequately covered in rural and low-income regions, where mental health support is already lacking.

To overcome these limitations, the field of behavioral medicine is gradually embracing data collected from mobile devices and is shifting toward a more personalized approach, better known as precision psychiatry. Recent advancements in precision psychiatry depend on the quantification of symptoms and treatment responses [1]. Multiple domains of biomarkers, such as physiological signals, brain imaging, and ‘omics’ molecular biosignatures, have been incorporated into conventional self-reported symptoms, clinical assessments, and neurocognitive batteries to yield the “big data” of the human brain [2].

It has not been long since novel technologies were first proposed as tools to trace people’s thoughts, feelings, and behaviors in a moment-by-moment manner. Bidargaddi et al. introduced the application of a “digital footprint” in psychiatry in 2017, which they defined as traceable data arising from people’s day-to-day activities on Internet-connected or mobile devices [3]. If properly analyzed, these human–machine interactions can be viewed as an objective proxy for humans’ multi-faceted mental activities. The richness of such data allows researchers to identify individual variations in digital phenotypes more precisely.

Other fields of research have also prospered due to the increasing availability of digital footprints. For instance, “cyberpsychology” is a discipline that studies the psychological phenomena that emerge in cyberspace, like internet addiction [4]. Many academic works in this field have revealed the potential psychological impact of social media, online games, and virtual reality technologies [5]. Meanwhile, stemming from the field of psychometrics, “psychoinformatics” utilizes digital footprints to collect big data at low costs and applies data mining techniques to investigate personal characteristics, including personality and mood states [6,7]. Lastly, derived from genetic phenotyping, the notion of “digital phenotyping” uses information retrieved from cell phones, smart watches, and the internet to demonstrate risky health behaviors and disease symptoms. From a clinical perspective, the digital phenotyping of patients may aid in the outcome prediction or severity assessment, which share the same vision as precision psychiatry [8].

## 2. The Strengths of Mobile Apps as Research Tools for Precision Psychiatry

Amid all the modern technologies that fuel the development of digital phenotyping, mobile applications (apps) have gained great popularity among mental health researchers, since they possess several advantages. Firstly, the ubiquity of smartphones in the present day ensures people’s accessibility to mobile apps across all nations [9]. Using apps to collect data also helps overcome the limitations associated with traditional research methods, such as when household surveys are too costly to be conducted in low-income communities or developing countries. Secondly, mobile apps serve as convenient research platforms for ecological momentary assessments. Users can be notified to complete survey questions at preset intervals or upon specific triggering events, such that evaluations can be made in real time in real-world settings with minimal memory bias [10]. In addition, Torous et al. suggested that conventional survey questionnaires tend to underestimate the severity of psychopathologies due to the Hawthorne effect, also called the observer effect, whereas assessments via mobile apps are more sensitive to depressive symptoms and suicide risk [11].

**Figure 1:**
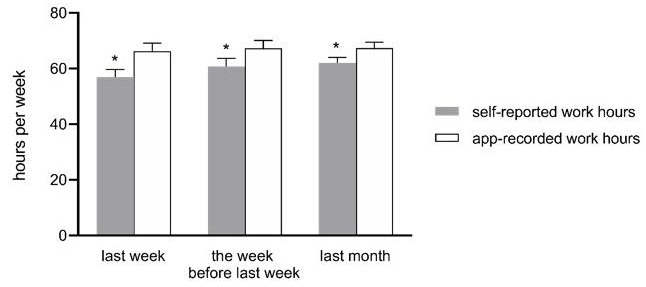
The bar graph compares self-reported work hours and app-recorded work hours at three time points in the past. The solid grey bar represents physicians’ self-reported weekly work hours, and the white bar represents the weekly work hours recorded by the “Staff Hours” app. * The asterisks indicate that self-reported work hours and app-recorded work hours had statistically significant differences. When asked to recall their work hours last month, physicians reported working for an average of 60.24 h per week, whereas the app recorded 66.94 h per week. In other words, physicians’ self-reported work hours showed a 11.5–12.0% reduction compared to their app-recorded work hours. In terms of work hours during the previous week, physicians underestimated their work hours by an average of 8.97 ± 8.60 h. Similarly, they underestimated their work hours the week before last week by an average of 6.48 ± 8.29 h. Physicians who worked for more hours underestimated their work hours to a greater extent compared to those who worked for fewer hours (r = −0.410, *p* = 0.013). The proportion of days in which physicians could not accurate recall their work hours increased as they tried to recall a more distant time in the past (r = 0.489, *p* = 0.002).

Another advantage to using mobile apps is that they can collect both passive and active data. The former refers to data generated without any direct involvement from the subject, such as Global Positioning System (GPS) traces and phone call logs, whereas the latter is defined as data that requires active participation from the subject, such as surveys and questionnaires [12]. Passive data are expected to better reflect users’ everyday behaviors and avoid recall bias, because the continuous data collection process occurs in the background and neither interrupts smartphone usage nor requires active input [13]. A study involving college students, for instance, reported that their average total time of weekly smartphone use was 20.11 ± 12.40 h based on self-reports but nearly 29.39 ± 14.45 h as recorded by mobile apps [14]. In another study, it was found that, when asked to recall the average weekly work hours within the past month, physicians reported working for an average of 60.24 h per week, despite their mobile apps recording 66.94 h per week [15]. Figure 1 illustrates that there was, on average, a 11.5–12.0% reduction in work hours reported by the subjects themselves. Further data analysis showed that the proportion of days in which physicians could not accurately recall their work hours increased as they tried to recall a more distant time in the past (r = 0.489, *p* = 0.002). All-in-all, these examples demonstrate how individuals tend to glamorize their behaviors and lifestyles due to the Hawthorne effect. In such scenarios, passive data collection by mobile apps can help minimize time distortion during subjective recall.

Lastly, mobile apps monitor mental activities with a higher temporal resolution than clinical interviews, phone calls, or household surveys can. With traditional research methods, psychopathologies such as insomnia, mood symptoms, or addictive behaviors are usually measured weekly, monthly, or even quarterly. This practice leaves the possibility that intermittent emotional turmoil or suicidal impulses may be missed between each observation [11]. Contrastingly, with the recent advancements in smartphone research techniques, intensive smartphone usage with rapid switches between social media, gaming, and productivity apps within short periods of time can now be accurately recorded via second-to-second passive data collection; these data can then be submitted for an analysis of problematic smartphone use [14]. By the same token, mobile apps designed to monitor sleep patterns provide precise measurements of sleep durations. This has enabled researchers to determine the sleep-reducing effect of smartphone use at night by the minutes, which may not be revealed through sleep diaries [16]. Lastly, sleep apps can differentiate the midpoints of sleep on weekdays from the midpoints of sleep during the weekends to calculate social jetlag [16].

## 3. Fundamental Principles of Behavioral Analysis Using Digital Biomarkers

**Figure 2:**
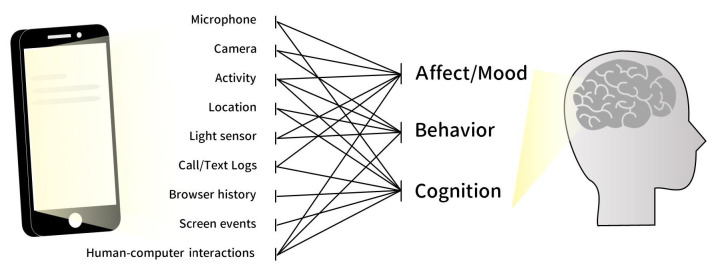
Smartphone hardware and software offer valuable sources of digital biomarkers that reflect users’ moods, behaviors, and cognitions at any point in time. These sources include microphones, cameras, activity and location monitors, light sensors, call or text logs, browser history, screen events, and human–computer interactions.

Smartphone hardware and software offer valuable sources of digital biomarkers that reflect users’ moods, behaviors, and cognitions at any point in time (Figure 2). These sources include microphones, motion sensors, human–machine interactions, and screen events [9,17]. For example, through speech processing, a user’s volume, intonation, and word choice can be analyzed to illuminate his or her mental state. By the same token, motion sensors record real-time locations, activity footprints, and social participation, which have direct implications on users’ mental activities. Likewise, human–machine interactions are a reliable biomarker that can show predictions highly correlated with the results from gold standard neurocognitive assessments, such as the symbol digit modality and digits backward test [18]. Utilizing these aforementioned biomarkers is timelier and more cost-effective than conducting conventional assessments, thus demonstrating a far greater potential for the early prevention, screening, and monitoring of cognitive impairments [18].

Previous studies on compulsive behaviors found that proactive smartphone use increased the risk of smartphone addiction, while reactive usage for the purposes of receiving emails, text messages, or calls did not account for problematic use [19]. The reason is that, unlike other addictive substances, smartphones themselves are not totally harmful to people. Instead, they play an important role in e-commerce and social networking, so it is impractical to detect problematic behaviors solely based on the duration of exposure. Consequently, it has been suggested that the connection between screen-on actions and notifications can help transform smartphone-centered phenomena into human-centered behaviors.

*Know Addiction* and *Menthal* on the Android [14,20], as well as *Screen Time* on the iPhone Operating System [21], are examples of mobile apps on the market that transform screen events into digital biomarkers. Generally, such apps collect three types of information: the timestamps of screen-on/off, the timestamps of notifications received, and the type of apps used. Once collected, the data is processed to construct a digital lifeline that can be used to interpret behavioral patterns and, in turn, predict whether a user is working or not.

**Figure 3:**
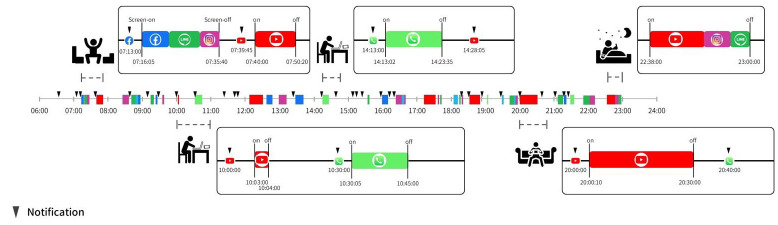
Apps designed to perform digital phenotyping automatically record smartphone events as three variables: (1) timestamps of screen-on/off, (2) type of apps used, and (3) notifications. The time between screen-on and successive screen-off is defined as one episode of use. The icons between screen-on and screen-off events represent the apps used during the episode. The information is processed by app algorithms to quantify the duration of app usage, the timestamps of notifications during and outside usage episodes, the reaction time from the moment a push notification is received to the start of app usage, and the reaction intensity, which is the duration of app usage following the notification. The digital lifeline illustrated in this figure spans from 6:00 in the morning to midnight; it specifically highlights the user’s contrasting usage behaviors during work and off-work hours.

Figure 3 illustrates a hypothetical user’s digital lifeline from 6:00 in the morning to midnight. The time between each screen-on and successive screen-off is defined as an episode of use [14]. This digital lifeline highlights that the length of episode, the type of apps used, the reaction time from the moment a push notification is received to the start of app usage, and the duration of app usage following the notification can be vastly different depending on the time of day. For example, the user receives a push notification from YouTube at 10:00. Upon seeing the notification, the user clicks into the YouTube app for only one minute due to being at work. At 10:30, the user receives a work call, which the user immediately responds to and engages in for 15 min. Later in the evening, the user receives another YouTube push notification at 20:00 and clicks into the app promptly. Being most likely off work at this point, the user leisurely spends 30 min on YouTube and misses a call at 20:40. As such, although the same two apps were involved from 10:00 to 11:00 and from 20:00 to 21:00, the usage patterns during each time range were dissimilar. This application of digital biomarkers highlights how smartphone usage patterns differ between work and off-work hours and, thus, can be used to shed light on the user’s mood, behavior, and cognition at any given moment in a day.

In regard to long-term behaviors and trends, Lin et al. suggested that digital biomarkers yielded by mobile apps can reliably unravel the erratic nature of mental activities with high temporal stability and accuracy [14]. Previous studies used an empirical mode decomposition analysis to examine the weekly variations and monthly intrinsic trends of excessive smartphone use based on daily use counts. Other studies have revealed that median usage lengths were more appropriate than mean durations to reflect trends in smartphone use [22,23]. Furthermore, Pan et al. showed that a continuous recording of smartphone use for two weeks could predict users’ behaviors up to eight weeks into the future [19]. This finding, in turn, provided a basic observation period for cyberpsychology and mobile health research.

## 4. The Application of Mobile Technologies in Mental Health Research

The development of digital biomarkers brought a paradigm shift to the way psychiatry is studied. According to the biopsychosocial model, an individual’s mental well-being is the result of a complex interaction between the biological, psychological, and social factors. In other words, environmental stressors like work hours and lifestyle habits like sleep patterns are as important as genetics in determining one’s mental health or ill health. In this regard, smartphone-obtained digital biomarkers help facilitate a deeper understanding of mental health and its pertinent labor policies. This idea can be explored from two approaches: (a) computing sleep–wake cycles and (b) tracking work hours.

### 4.1. Computing Circadian Rhythms and Sleep Patterns via Smartphones

Objectively detecting circadian disruptions and recording sleep patterns in the long term have been major challenges for scholars and clinicians for several reasons. Firstly, conducting in-laboratory polysomnography is labor-intensive, time-demanding, costly, and therefore unscalable for a large population [24]. Secondly, wrist-worn devices currently on the market are not widely accessible and have yet to attain the accuracy necessary to quantify sleep times. They also pose an inconvenience, requiring users to remember to wear the devices and keep them on for continuous cycles of sleep–wake recordings.

Compared to the standard polysomnography and wrist-worn devices, using mobile apps to measure sleep is relatively unobtrusive, given the widespread use and deep reach of smartphones in day-to-day life. However, most of the currently available apps must be manually set, and users must place their mobile devices next to their bedside to accurately monitor and record sleep patterns. If users forget to set or properly place their devices, the collected data will be rendered unusable. Meanwhile, there exist other apps on the market that utilize phone sensors to detect sound, light, and movement in the surroundings. However, those parameters are vulnerable to environmental disturbances and reflect users’ physical activities more so than their mental activities. Furthermore, using sensory-based algorithms typically requires high power consumption, which is unideal from the users’ standpoint.

Considering these obstacles, Lin et al. proposed a mobile app called “Rhythm” that could delineate proactive smartphone screen-on and screen-off patterns using low power consumption. The app revolutionized the idea of human–smartphone interactions as a proxy for circadian rhythms, as it could automatically estimate the sleep time and evaluate the circadian patterns in naturalistic settings with a 90.4% accuracy [25].

**Figure 4:**
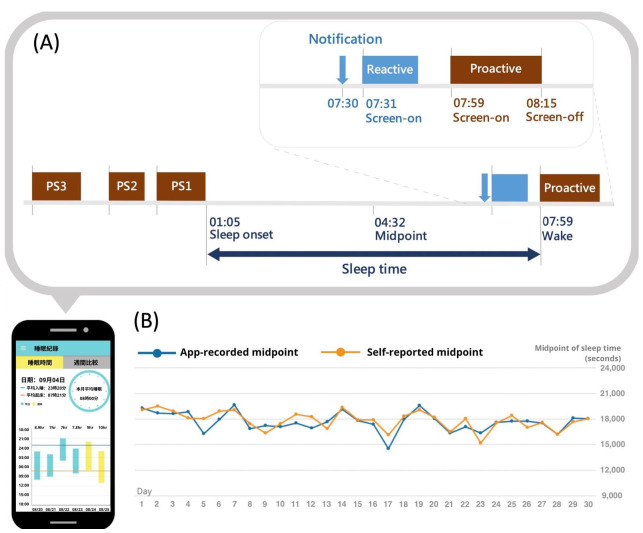
(**A**) Smartphone use from screen-on to the successive screen-off was defined as one episode of use. Usage episodes without notifications during the one minute before screen-on were classified as proactive use, whereas episodes with notification during the one minute before screen-on were classified as reactive use. Only proactive use episodes were included in the calculation of sleep time. (**B**) Daily app-recorded and self-reported midpoints of sleep time were compared to validate the fluctuations in circadian rhythms. The figure showed a high agreement between app-recorded and self-reported midpoints of sleep time.

Using “Rhythm”, Lin et al. found that smartphone use before bedtime has been linked to delayed bedtime and reduced sleep times. Even though the duration of smartphone usage before bedtime only accounted for approximately 14.3% of the total daily use, this relatively short period of screen time constituted nearly half of the daily impact on sleep [26]. In addition, this app chronicled users’ midpoints of sleep, which provided insights into social jetlag, as well as other subtle shifts in sleep patterns (Figure 4) [16]. These were a few consequential findings that would have remained undetected via conventional sleep studies or sleep recording tools. Beyond research purposes, the sleep parameters generated from “Rhythm” were applicable from a clinical standpoint as well. Specifically, chronic circadian dysregulation has been implicated in the increased risk of bipolar disorder, major depression, and schizophrenia [27]. Therefore, this app offered a personalized channel for the early detection and prevention of those mental disorders.

### 4.2. Automatically Recording Work Hours

Long work hours contribute to psychological stress, which can influence the onset and progress of health problems [28]. Medical professionals such as trainee physicians, for instance, have notoriously demanding clinical duties that cause them to work overtime on a regular basis. However, their prolonged work hours have been linked to higher risks of suicide, burnout, and heart diseases [29]. Being overworked also jeopardizes the quality of patient care [26]. Several countries have implemented work hour limits in response to this occupational health issue. Yet, it is difficult to accurately survey and supervise work hour compliance during physicians’ daily practice. One of the many reasons is that traditional labor surveys for the general population rely on self-reported average work hours, which fail to reflect work fluctuations among healthcare personnel with irregular work schedules and unpredictable on-call duties. Self-reporting is also impractical for defining work times at multiple institutions or at home.

As of 2019, there were about 500 mobile apps aimed at tracking work hours in the Google Play Store and iPhone Operating System App Store. However, most of them require users to manually clock in and out due to the apps’ lack of an automatic geofencing feature. Chiang et al. overcame this fundamental limitation by designing “Staff Hours”, an app that automatically chronicles users’ work patterns via a highly accurate, yet power-saving, GPS-defined algorithm [30]. Being the first of its kind, “Staff Hours” was specifically designed to monitor overwork patterns and occupational burnouts among medical staffs. Since the app runs in the background of smartphones, it places no additional burden on users beyond their typical interactions with their mobile devices.

**Figure 5:**
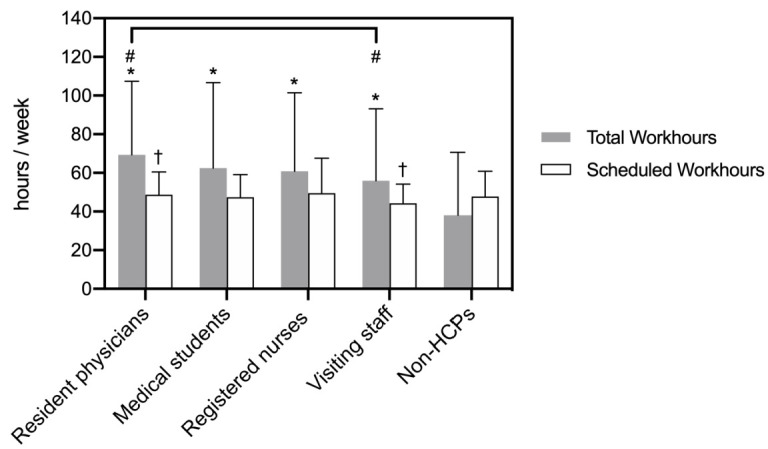
The bar graph compares the total work hours and scheduled work hours between five occupational categories. There were significant differences between the five categories of occupation (*p* = 0.003). Post hoc comparisons further revealed that all medical staff had significantly longer total work hours (resident physicians: 69.40 ± 38.03, *p* < 0.001; medical students: 62.50 ± 44.18, *p* = 0.020; registered nurses: 60.83 ± 40.62, *p* = 0.040; visiting staff: 55.89 ± 37.25, *p* = 0.041) than non-healthcare professionals (38.12 ± 32.51), as indicated by the asterisks *. In addition, resident physicians had statistically significantly longer work hours than visiting staff, both in terms of total work hours (represented by #) and scheduled work hours (represented by †).

Using aggregated datasets collected by “Staff Hours”, researchers can compare work hours between different types of healthcare personnel, as well as among workers of vastly disparate occupations. Figure 5 shows that medical staff had longer work hours than nonhealthcare professionals, and resident physicians worked longer hours than visiting staff in hospitals [30]. With the inclusion of regulations pertaining to resident physicians’ work hour limits in Taiwan’s Labor Standards Act in September 2019, this app took on another purpose in labor negotiations, serving as a real-time monitor for work hour limit compliance and evidence for employees when they claim overtime pay.

## 5. Challenges and Ethical Considerations

Several technical challenges should be noted when interpreting digital biomarkers delineated from mobile app data. Firstly, the iPhone Operating System and Android have vastly different app permissions. The latter has been adapted to different manufacturers’ devices, leading to many more variations of Android-formatted data in circulation compared to the iPhone counterpart. As such, cross-platform applications should be developed in the future to improve generalizability. Secondly, striking a balance between data volume and power consumption is a major barrier, because the rate at which mobile devices collect GPS data directly impacts the battery performance. Although a higher sampling rate is necessary to enhance temporal resolution, a higher power consumption is not ideal from the user’s standpoint. Thirdly, to address the challenge of low app retention rates, apps should increasingly move toward automated passive data collection rather than relying on active data input. This was exemplified by a study on “PTSD Coach”, an app developed for post-traumatic stress disorder (PTSD) management [31]. In this study, researchers found that active data collection in the app declined over time, from 10 to 12% of users filling out questionnaires one week after app installation down to 4 to 5% after one month. However, passive data collection did not drop as drastically, only decreasing to 27.3% after one month [31]. This suggests that continuously collecting passive data via background apps is less obtrusive and more advantageous for app retention.

From an ethical standpoint, data privacy is an unavoidable concern when working with apps that retain highly personal data. During the ongoing COVID-19 pandemic, for instance, the rates of depression and anxiety disorders have skyrocketed, facilitating more people in embracing online therapy services [32]. However, this boom in mental health apps has unveiled how a small negligence in privacy policy, such as failing to anonymize or encrypt patient notes, can lead to irreversible data breaches [33]. By the same token, information as simple as the timestamps of screen-on or -off events are sufficient to discern digital phenotypes between individuals. Therefore, human–smartphone interaction data should only be accessible to pertinent researchers and be stored with specifications equivalent to those used to safeguard personal health information, so as to uphold information security and research ethics.

With ethical considerations at the forefront, mental health professionals should prudently screen the mobile apps they recommend to patients. This entails validating the credibility of app developers by reading app reviews, avoiding apps primarily aimed at advertising products, and understanding the privacy policies on developers’ websites [34]. According to the American Psychiatric Association’s Ethics Committee, confidentiality, beneficence, and truthfulness are key ethical obligations [35]. In other words, clinicians should clarify what data will be collected, how information will be secured, and whether the benefits of use outweigh any possible harms. Most importantly, clinicians and patients should ensure that their goals in using mobile technologies align, so as to not compromise the trust between the doctor–patient relationship [35]. At the present time, efforts are already underway by international organizations to develop official guidelines that enforce transparency in data storage, use, and sharing practices [35]. All-in-all, with the digital footprint becoming an increasingly mainstream biomarker in medical research, ethical controversies surrounding technology use in healthcare will continue to generate heated discussions.

## 6. Conclusions

The development of digital biomarkers is highly relevant to the present landscape of psychiatry, offering novel modes of data collection. Electronic devices that can record human–machine interactions have provided researchers with an opportunity to overcome the limitations associated with conventional research tools. Smartphones, which are highly portable and accessible to both the rich and the poor, have received great attention for their ability to record passive data automatically and continuously in various forms, including screen-on and screen-off events, voices, motions, and types of apps used. Identifying the duration and frequency, proactive versus reactive, and chronicity and periodicity of smartphone use allows us to explore human mental activities. Examples from several research fields demonstrate how mobile apps transform human–machine interactions into digital biomarkers of mental illness.

## 7. Future Directions

Smartphones are not only integral digital companions in people’s lives nowadays but also show promising applicability to psychiatric research. With that said, the future of digital phenotyping is progressing in two main directions: (a) integrating human-smartphone interaction data into artificial intelligence evaluations, as well as (b) using digital biomarkers to offer personalized treatments and interventions.

### 7.1. Personalized Assessment—Digital Biomarkers for Artificial Intelligence Evaluation

Among the many potential applications of artificial intelligence (AI), its application in modern medicine is perhaps the most ground-breaking and promising. AI-based medical software can extract meaningful information and diagnose health complications, sometimes with greater precision than humans can. For example, Apple Watch received clearance from the United States Food and Drug Administration in 2018 for detecting atrial fibrillation among users.

At the same time, however, implementing AI technologies in a clinical setting has its challenges as well. With medical imaging, researchers need massive amounts of imaging data and clinician input to effectively train machine learning models and, in turn, ensure that they can correctly deliver diagnoses. In other words, the present obstacle comes down to the lack of labeled radiology datasets for AI algorithms to process.

On the other hand, the use of passive data collected via smartphones overcomes this fundamental issue. This is possible, because digital biomarkers are intrinsically annotated. Moreover, since mobile apps are capable of chronicling everything users see and do on their smartphone screens, there will not be a shortage of such data for training machine learning algorithms. In fact, the abundance of digitally obtained data has led to the creation of the “Human Screenome Project”, which highlights that screen events are as plentiful and informative as human genomic data [36]. Furthermore, passive data are continuously collected in the background of smartphones without necessitating active input, which enables highly complex behaviors to be assessed with minimal recall bias [13].

**Figure 6:**
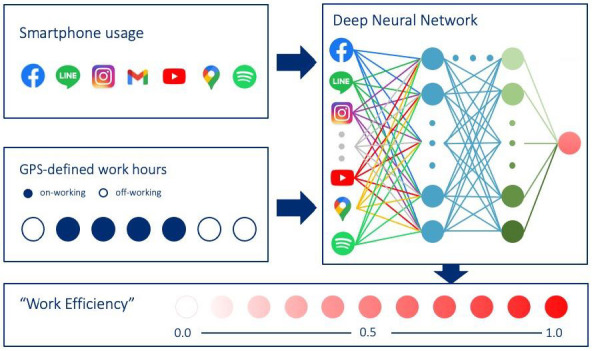
The deep learning model is trained by smartphone usage patterns and the corresponding GPS-defined working status. Smartphone usage patterns are formulated as a time series of screen-on/off, notifications, and types of mobile apps. The GPS-defined working/off-working statuses are labeled as 1 or 0. We will train a multilayer deep neural network and tune the weighing of the model. The trained deep learning model yields a probability between 0 and 1 from any time series of the smartphone usage patterns. This working probability can be interpreted as the work efficiency.

Figure 6 visualizes a paradigm, in which a deep learning model is trained by smartphone usage patterns and the corresponding GPS-defined working status to evaluate the user’s work performance. Smartphone usage patterns are recorded based on the timestamps of screen-on/off, types of apps used, and notifications. Meanwhile, GPS-defined work statuses are labeled as either 1 or 0. Given the characteristics of smartphone usage patterns, either convolutional neural networks (CNNs) or recurrent neural networks (RNNs) are proposed to generate working probabilities that reflect work efficiencies. The rationale behind selecting those two neural network models is that the former has previously been used in medical imaging, while the latter has been used in natural language processing. Both of these fields are concerned with the data of similar characteristics as those of digital biomarkers from smartphone usage patterns. More specifically, screen events are chronicled as a sequence of timestamps in the same way languages are coded as linguistic sequences for RNN modeling. Meanwhile, screen events also offer adequate temporal resolution for a feature analysis in the same way radiology images can be processed via CNN. All-in-all, this paradigm paves the way for further research on relevant deep learning model applications in human activity recognition.

**Figure 7:**
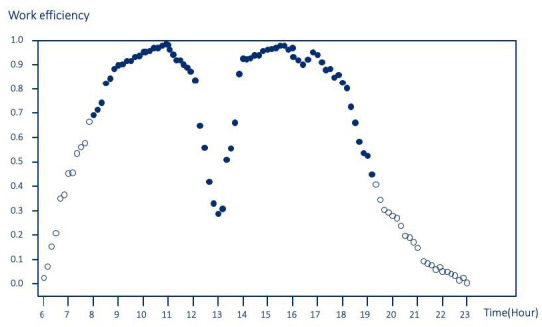
The scatterplot illustrates the proposed “work efficiency” diagram for one day. The sold dots represent GPS-defined work hours, whereas the hollow dots represent the GPS-defined off-work period. In this scenario, the individual’s work hours start at 8:00 and end at 18:00. The individual’s “work efficiency” is greater than 0.90 between 9:00 and 12:00, as well as between 15:00 and 17:00. The lowest “work efficiency” occurs at around 13:00; at which point, the efficiency level is similar to those during off-work hours.

When plotted against the hours in a day, it becomes clear when efficiency peaks and troughs are. For example, considering an individual whose work hours start at 8:00 and end at 18:00, the “work efficiency” is the highest between 9:00 and 12:00, as well as between 15:00 and 17:00 (Figure 7). During the lunch break, the “work efficiency” drops to a level similar to those during off-work hours. This index is also useful when studying the health and behaviors of individuals with more complicated work schedules. For instance, healthcare workers frequently have on-call duties and can sometimes be assigned to work at multiple locations. In this case, using AI to track their work hours and to validate overtime may allow for a better compliance with labor regulations, as well as better work–life balance in the long term.

### 7.2. Personalized Intervention—Digital Therapeutics

Beyond diagnosing diseases, digital biomarkers serve therapeutic purposes as well; this includes encouraging patients to adhere to medication regimens, diet plans, and exercise routines [37]. For example, an American digital therapeutics company has taken the novel approach of integrating mobile apps with ingestible sensors and skin patches to overcome poor medication adherence [37]. The ingestible sensors are embedded into pills that are coated with magnesium and copper on either side. Once a pill is swallowed, the two elements combine, causing the sensor to signal a patch worn on the patient’s skin. The patch can then be linked to a mobile app on the patient’s smartphone, which enables data sharing with healthcare providers given that the patient has consented. The patch can also monitor the patient’s activity, heart rate, sleep quality, and temperature, thereby offering other measurements of the patient’s response to the medication [37].

**Figure 8:**
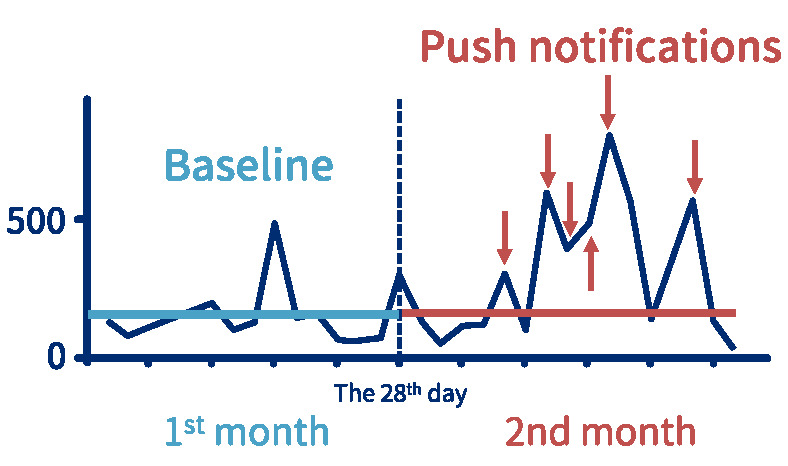
Smartphone apps can act as personalized reminder systems that intervene in excessive smartphone use. For example, the “Know Addiction” app automatically calculates users’ average smartphone usage time over one month and sets it as a personalized benchmark (baseline). If the usage exceeds the benchmark at any point during the following month (red arrows), a push notification is sent out to advise the user to reduce their usage and smartphone dependence.

In terms of cognitive–behavioral therapy, several apps have been developed to address mental health disorders. For instance, in a randomized controlled trial involving 5800 individuals in the process of smoking cessation, participants in one study group received automated text messages reminding them to quit smoking while the other group did not. After six months, those who received reminders significantly improved in their smoking cessation rates [38]. Similarly, A-CHESS is an app designed to improve the continuing care for alcohol use disorders, and its implementation reduced the number of risky drinking days reported by patients [39]. In terms of uncontrollable smartphone use, an app called “Know Addiction” automatically calculates users’ average smartphone usage time over one month and sets it as a personalized benchmark. If the usage exceeds the benchmark at any point during the following month, a push notification is sent out to advise the user to reduce usage and smartphone dependence (Figure 8). All of the aforementioned examples demonstrate how smartphones can provide personalized and scalable behavior intervention and therapy in a timely manner.

In summary, the fast-paced evolution of digital biomarkers has brought forth a new era of psychiatry, one that seeks diagnoses and interventions that are scalable yet tailored to each individual’s needs. With more than three billion smartphone users worldwide in the present day, smartphones may be the most promising tool to make a difference in those suffering from chronic mental illnesses for which existing face-to-face treatments do not always work. Once matured and implemented to its full capacity, digital biomarkers can enhance the quality of mental healthcare received by patients, as well as increase treatment accessibility globally.

## Data Availability

Not applicable.
